# Succinate ameliorates mitochondrial oxygen consumption of metformin-intoxicated human platelets

**DOI:** 10.1186/cc12389

**Published:** 2013-03-19

**Authors:** A Protti, M Monti, A Lecchi, A Artoni, N Greppi, L Gattinoni

**Affiliations:** 1 Fondazione IRCCS Ca' Granda Ospedale Maggiore Policlinico, Milan, Italy; 2 Università degli Studi di Milano, Milan, Italy

## Introduction

Metformin intoxication inhibits mitochondrial complex I and oxygen consumption (VO_2_). Succinate bypasses complex I by donating electrons to complex II. The aim of this study was to clarify whether succinate ameliorates mitochondrial VO_2 _of metformin-intoxicated human platelets.

## Methods

Platelet-rich-plasma was incubated for 72 hours with metformin at a final concentration of 0 mg/l (control), 1.66 mg/l (therapeutic dose) or 166 mg/l (toxic dose). Platelet VO_2 _was then measured with a Clark-type electrode, in the presence of glutamate plus malate (complex I electron donors) (final concentration: 20 mmol/l for both) or succinate (complex II electron donor) (30 mmol/l), before and after adding cyanide (40 mmol/l). Mitochondrial (cyanide-sensitive) and extra-mitochondrial (cyanide-insensitive) VO_2 _were corrected for platelet count.

## Results

**Figure 1:**
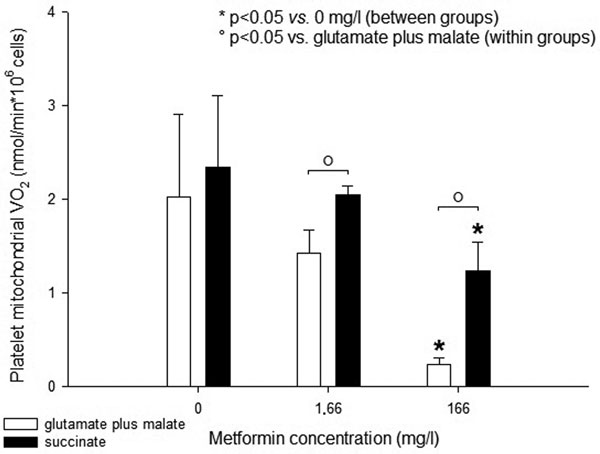
**Mitochondrial oxygen use of human platelets incubated with metformin**.

The main results, from four preliminary experiments, are shown in Figure 1. In the presence of glutamate plus malate, only platelets incubated with a high dose of metformin had a mitochondrial VO_2 _significantly lower than controls. In the presence of succinate, mitochondrial VO_2 _of controls did not change significantly whereas that of platelets incubated with metformin did. The effect of succinate tended to become larger as the dose of metformin was increased from 0 up to 166 mg/l (0.3 ± 0.2 vs. 0.6 ± 0.3 vs. 1.0 ± 0.3 nmol/minute*10^6 ^cells) (*P *= 0.068).Even so, mitochondrial VO_2 _of platelets incubated with.the highest dose of metformin did not return to the levels of controls. Extra-mitochondrial VO_2 _was always the same.

## Conclusion

Succinate ameliorates (but does not return to normal) mitochondrial VO_2 _of human platelets incubated with a toxic dose of metformin.

